# Case Report: Lumbar herniation of a low-grade appendiceal mucinous neoplasm mimicking a chronic abscess

**DOI:** 10.3389/fonc.2025.1674059

**Published:** 2026-01-05

**Authors:** Wei Gao, Yifan Feng, Gang Liu, Yefei Sun, Jianping Zhou

**Affiliations:** 1 Department of Gastrointestinal Surgery, The First Hospital of China Medical University, Shenyang, Liaoning, China; 2 Shenyang Medical Nutrition Clinical Medical Research Center, Shenyang, Liaoning, China

**Keywords:** low-grade appendiceal mucinous neoplasm (LAMN), lumbar hernia, colonic diverticular disease, case report, chronic abscess

## Abstract

Low-grade appendiceal mucinous neoplasms (LAMNs) are uncommon tumors that may lead to peritoneal dissemination if not completely excised, yet they often present with nonspecific symptoms. We describe a 53−year−old male with a three−year history of recurrent right lumbar “abscesses” whose contrast−enhanced CT suggested herniation of the ileocecal region into the abdominal wall. Laparoscopic exploration revealed a superior lumbar hernia containing an enlarged appendix with surrounding purulent fluid. The patient underwent laparoscopic ileocecal resection with side−to−side ileocolic anastomosis and drainage. Postoperative pathology confirmed a LAMN with surrounding suppurative changes and diverticular features in the right colon. No mesh repair was performed due to active inflammation; instead, staged hernia repair was planned. The patient recovered uneventfully, with no evidence of pseudomyxoma peritonei or hernia recurrence at follow−up. This case highlights the importance of considering appendiceal neoplasia in atypical hernias mimicking chronic abscesses, as timely recognition and complete oncologic resection are critical to prevent mucinous peritoneal spread and achieve optimal outcomes.

## 1 Introduction

Lumbar hernia is an uncommon abdominal wall hernia with variable clinical presentations, often leading to diagnostic delays. Appendiceal herniation into the lumbar region with secondary abscess formation is exceptionally rare, with few cases reported. Moreover, low-grade appendiceal mucinous neoplasms (LAMNs) are an uncommon subset of appendiceal tumors characterized by mucinous epithelial proliferation confined to the appendix; if not recognized and completely resected, LAMNs carry the risk of peritoneal dissemination and pseudomyxoma peritonei, a serious oncologic complication. We present this rare case of a LAMN herniating into the superior lumbar triangle (Grynfeltt’s hernia), mimicking a chronic abscess, and discuss its diagnostic challenges, laparoscopic management, and oncologic implications for preventing mucinous peritoneal spread.

## 2 Patient information

A 53-year-old Han Chinese male from Tieling, Liaoning Province, presented with a three-year history of right-sided lumbar pain, exacerbated over the past two weeks. He was married and worked in the industrial sector. There was no history of trauma, fever, vomiting, or altered bowel habits. Three years prior, he underwent percutaneous drainage of a right lumbar abscess (~500 mL pus aspirated), followed by recurrent abscess formation treated with three additional drainage procedures. Recent symptom progression with radiating lumbar pain prompted referral to our hospital.

## 3 Past medical history

No history of cardiovascular disease, diabetes, or drug allergies. The patient has smoked approximately 20 cigarettes daily for 30 years (≈30 pack-years) and reports daily consumption of about 250 mL of white spirit for the past 10 years. No family history of relevant diseases.

## 4 Clinical findings

Physical examination revealed a soft, tender, and mobile 3 × 4 cm mass in the right lumbar region, non-reducible in the supine position, without overlying erythema or warmth. Bowel sounds were audible on auscultation.

## 5 Timeline of events

The timeline of the patient’s presentation and management is shown in **Table 1**.

**Table 1:** Key events from initial presentation to discharge.

Date	Event	Details
3 years ago	Initial right lumbar abscess	Underwent percutaneous drainage
Recurrent over the past three years	Recurrent lumbar abscesses over the past three years	Total of 3 drainage procedures across this period
2025-05-23	Worsening pain with back radiation	Symptom worsening ~2 weeks before diagnosis (approx.)
2025-06-06	Diagnosis	Perforated cecal diverticulum and lumbar abscess
2025-06-11	Surgery	Laparoscopic ileocecal resection
2025-06-17 (Postoperative day 6)	Discharge	Recovery satisfactory; discharged in stable condition
2025-09-10	Follow-up examination	No symptoms of lumbar hernia recurrence or bowel dysfunction

## 6 Diagnostic assessment

**Figure 1:**
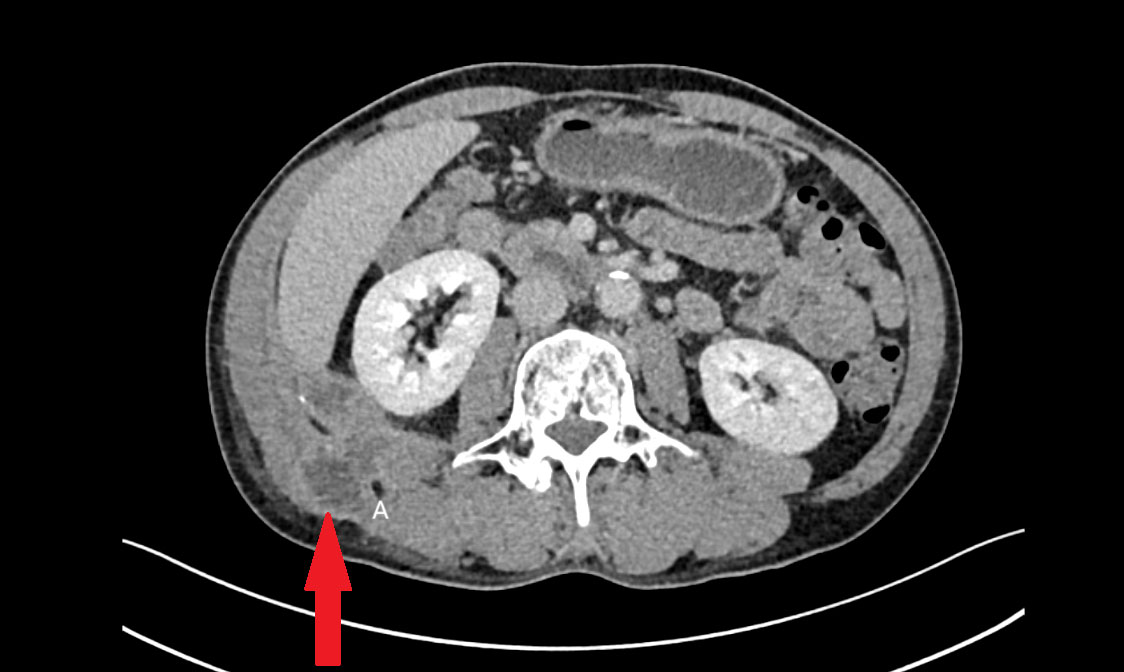
Contrast-enhanced CT showing the abscess in the abdominal wall. The abscess is indicated by the red arrow.

**Figure 2:**
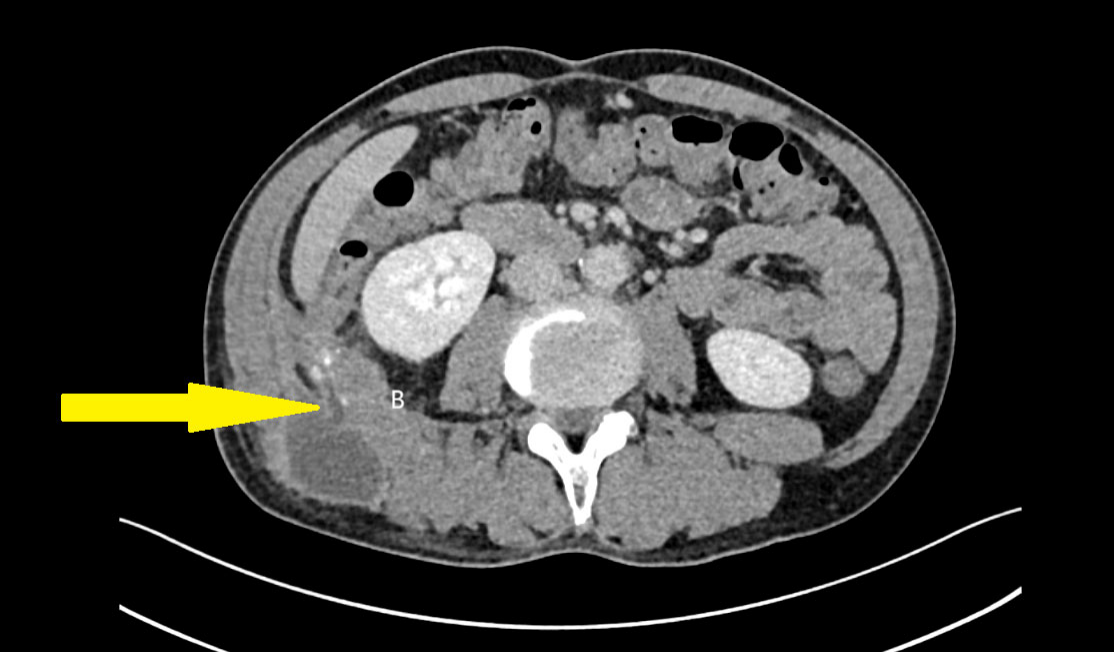
Contrast-enhanced CT showing herniation of the cecum and appendix through the abdominal wall into the lumbar region. The cecum is indicated by the yellow arrow.

Admission diagnosis: “Perforated right cecal diverticulum with associated lumbar abscess.” Contrast-enhanced CT scan revealed herniation of the ileocecal segment through the right external oblique and iliocostal muscles into the abdominal wall, with a cystic outpouching suggestive of a diverticulum. Other abdominal structures appeared normal. The chronic, recurrent nature of the abscess led to initial misdiagnosis as subcutaneous infection. Concurrently, the inflammatory edema secondary to the abscess obscured the anatomical tissue planes and blurred the imaging distinction between the abscess cavity and the appendix, preventing its definitive localization preoperatively (**Figure 1**, **2**).

## 7 Therapeutic intervention

**Figure 3:**
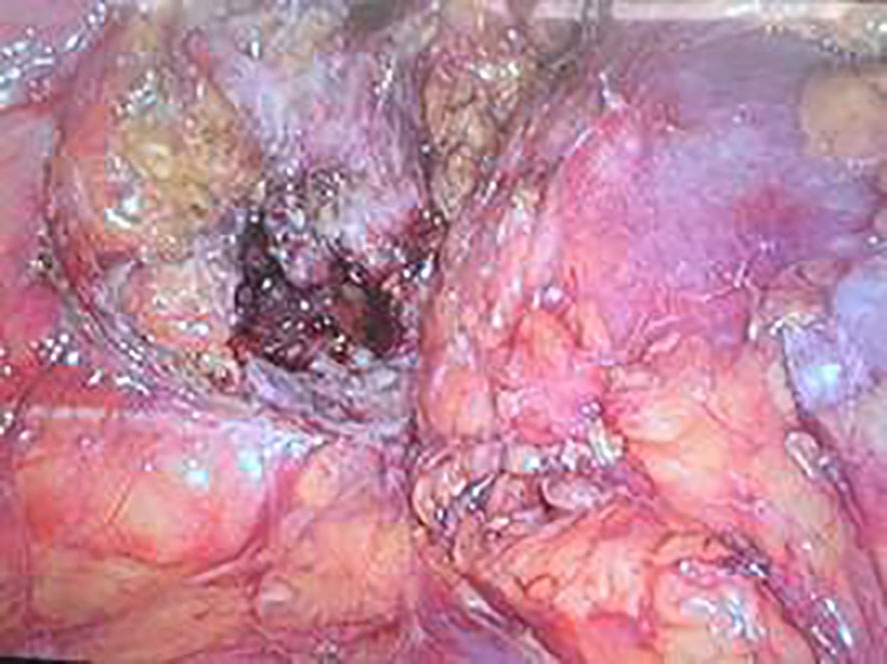
Laparoscopic view of the lumbar hernia, with marked edema of the surrounding abdominal wall; the herniated segment of the cecum is seen adjacent to the hernia orifice.

**Figure 4:**
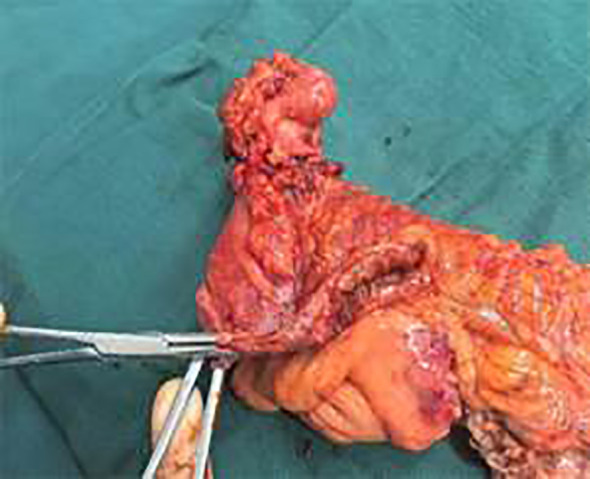
Gross specimen of the resected ileocecal segment, demonstrating the cecal structure and the inflamed, contracted appendix invaginated into the cecal lumen.

Laparoscopic exploration under general anesthesia revealed a lumbar hernia below the right kidney. The ileocecal region and the appendix were contained within the lumbar hernia sac, with the terminal ileum adherent to the right lower abdominal wall. The right half of the greater omentum was resected. The gastrocolic ligament was divided lateral to the gastroepiploic vascular arcade and the dissection was extended to the anterior aspect of the pancreatic head, mobilizing the transverse mesocolon and the hepatocolic ligament. At a point 1 cm superior to the iliac vessels, the ileocecal mesentery was incised and dissection was advanced upward and to the right into Toldt’s space; the dissection was then carried cranially along the anterior renal Gerota fascia to the lateral peritoneum of the ascending colon and the hepatocolic ligament. Incision of the hernia sac released purulent fluid. Further dissection showed the appendix herniated into the sac, with surrounding bowel wall edema and thickening. After reduction of the appendix and a segment of the cecum back into the peritoneal cavity, the lumbar hernia defect was clearly delineated. (**Figure 3**) The ileocolic artery and vein were ligated and divided. The mesentery of the ascending colon was incised superiorly up to the hepatic flexure of the transverse colon. The bowel was mobilized and the mesenteric attachments were skeletonized, and the transverse colon was divided using a linear cutting stapler. A 6-cm midline abdominal incision was made and the right colon was exteriorized; the ileum was transected 20 cm proximal to the ileocecal valve. A side-to-side anastomosis was fashioned between the ileal limb and the transverse colon, and the common enterotomy was closed with a continuous inverting seromuscular suture. Two drains were placed: one at the anastomotic site and one within the hernia cavity to ensure adequate drainage. The gross appearance of the resected ileocecal specimen is shown in **Figure 4**. Owing to pronounced inflammatory edema of the surrounding abdominal wall, we did not perform a primary hernia defect closure with mesh. Instead, we opted for thorough drainage and planned a staged lumbar hernia repair.

## 8 Follow-up and outcomes

**Figure 5:**
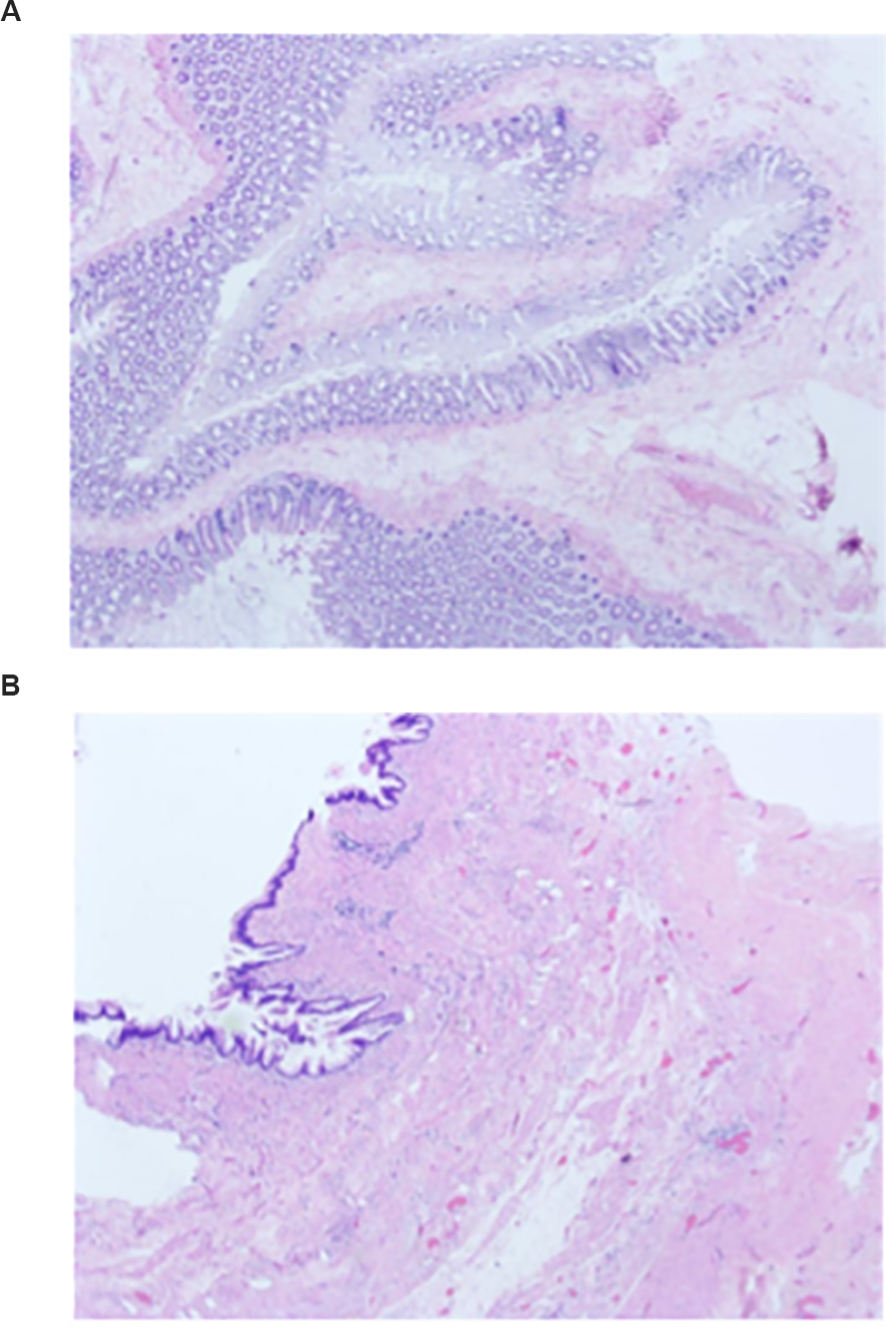
**(A)** Hematoxylin–eosin–stained section of the appendiceal lesion showing mucin-producing epithelium with low-grade cytologic atypia and abundant extracellular mucin, accompanied by focal suppurative inflammatory change. **(B).** Hematoxylin–eosin–stained section of the appendiceal wall showing abundant extracellular mucin dissecting into the mural layers with an accompanying fibrotic reaction and chronic inflammatory cell infiltration.

On postoperative day 1 the patient tolerated oral fluids. On postoperative day 3 the patient advanced to an oral elemental diet and resumed normal physical activity. On postoperative day 6 the intra-abdominal drain placed posterior to the ileocolic anastomosis evacuated 60 mL of ascites-like serous fluid, while the drain within the abdominal wall hernia sac yielded 2 mL of lightly blood-tinged fluid; and both drains were removed. During follow-up, the patient remained asymptomatic with no evidence of disease recurrence. The postoperative pathology report confirmed a low-grade appendiceal mucinous neoplasm with surrounding suppurative changes; examination of the resected right colon specimen demonstrated features consistent with colonic diverticula (**Figures 5a**, **b**). All regional lymph nodes in the surgical specimen were examined histopathologically and showed no evidence of tumor metastasis. Both the proximal (ileal) and distal (colonic) resection margins were free of tumor, consistent with an R0 resection. Based on these findings, no additional treatment was indicated, and the patient continues to be monitored at regular intervals. At the three-month postoperative follow-up, the patient reported satisfactory recovery of bowel function. Physical examination of the lumbar region revealed no tenderness or palpable mass. The decision not to perform definitive lumbar hernia repair at the index operation was intentional: because of pronounced inflammatory edema and intra-operative contamination of the hernia sac, primary mesh repair was considered unsafe and a staged repair was planned after infection control. At the three-month postoperative follow-up the patient remained asymptomatic, in good general condition, and satisfied with his current status. Therefore, the patient will continue under periodic surveillance for potential late recurrence or development of hernia-related symptoms.

## 9 Discussion

Lumbar hernias are rare and often misdiagnosed as lipomas or cold abscesses due to atypical presentations (1). They occur in weakened zones between the 12th rib, iliac crest, and paraspinal muscles, classified as superior (Grynfeltt’s triangle) or inferior (Petit’s triangle) lumbar hernias (2, 3). This primary lumbar hernia remained undiagnosed for years until imaging revealed intra-abdominal content herniation. Appendiceal involvement in hernias accounts for only ~1% of abdominal wall hernias, predominantly in inguinal (Amyand’s hernia) or femoral (de Garengeot’s hernia) location (4, 5). Lumbar hernias are uncommon, with only a few hundred cases described in the literature historically. Although appendiceal involvement is well-recognized in other abdominal wall hernias (for example Amyand’s hernia in the inguinal region and de Garengeot’s hernia in the femoral canal), appendiceal herniation into the lumbar region is exceedingly rare and has been reported only as isolated case reports. A small number of case reports have documented herniation of the ileocecal region into lumbar hernia sacs. Rubén Neris et al. described a 69-year-old man who sustained a traumatic lumbar hernia after a motor-vehicle collision, with the cecum and ileocecal valve incarcerated at the iliac crest, consistent with an inferior lumbar hernia (6). Eric Bergeron et al. reported a 67-year-old woman with no prior abdominal surgery whose appendix herniated into the right iliac fossa and subsequently perforated, resulting in necrotizing fasciitis of the flank (7). Max Scheffler et al. described a 92-year-old woman in whom a segment of the ascending colon protruded through the posterior abdominal wall; the hernial sac was located immediately inferior to the right 12th rib, and the proximal cecum and small-bowel loops were dilated, producing clinical and radiological features of intestinal obstruction (8).

Lumbar hernias rarely contain retroperitoneal structures like fat, colon, or kidney (9). Anomalous appendiceal positions—such as retrocecal or post-cecal, especially with chronic inflammation—may lead to abscess formation and secondary lumbar herniation (10). A mobile cecum (10–20% prevalence) or congenitally short cecum predisposes to ectopic appendix position (11, 12). Failure of cecal fixation can displace the appendix into atypical sites such as the subhepatic region or lumbar area (13, 14).

Infected hernias with appendiceal abscesses are not suitable for primary mesh repair due to infection risk. A staged approach—initial infection control with appendectomy and drainage, followed by elective hernia repair—is recommended (10), mirroring management of Amyand’s hernia with abscesses (15). Drainage and delayed mesh repair are favored in infected lumbar hernias (16).

Oncologic Considerations of Low−Grade Appendiceal Mucinous Neoplasms (LAMNs): Low-grade appendiceal mucinous neoplasms feature low-grade mucinous epithelium, often with villiform, undulating, or flat architecture, and extend by mimicking diverticula (17). Surgeons should maintain a high index of suspicion for neoplastic processes in atypical hernia contents, particularly when gelatinous or mucinous material is encountered intraoperatively. LAMN is an uncommon entity, accounting for approximately 0.4% to 1% of all gastrointestinal malignancies in the United States (18). Reported incidence varies between populations and regions: a German study estimated an incidence of approximately 0.13%, whereas Smeenk et al. reported an incidence of 0.52% in a Dutch cohort (19, 20). To our knowledge, there are no previously reported cases of LAMN presenting as the content of a lumbar hernia, underscoring the rarity and reporting value of the present case. LAMN is generally slow-growing and of low malignant potential. While the majority of patients do not experience recurrence following appendectomy or limited cecectomy, a subset may progress to pseudomyxoma peritonei (PMP) (21). PMP is characterized by diffuse intraperitoneal mucinous dissemination and can be life-threatening (22). Reported overall postoperative recurrence rates are approximately 6% (23). Risk factors associated with recurrence or progression to PMP include histologic evidence of mucin or tumor cells breaching the serosa, appendiceal wall fibrosis with mucin infiltration, separation of mucinous masses from the primary tumor, positive intraoperative or postoperative resection margins, postoperative elevation of tumor markers (CEA, CA19-9, CA-125), and AJCC M staging of M1b. These features have been associated with increased risk of disease progression and adverse outcomes (23–25). The principal staging systems applied to LAMN are the American Joint Committee on Cancer (AJCC) staging and the Peritoneal Surface Oncology Group International (PSOGI) classification (26). Because LAMN characteristically demonstrates an expansile (“pushing”) growth pattern rather than conventional infiltrative adenocarcinoma, T staging focuses on whether mucin or tumor cells extend through the muscularis and involve the serosa or adjacent structures. Specifically: pTis indicates tumor confined to the appendiceal wall, with mucin or epithelial lesions present within the muscularis but without penetration—this stage carries a favorable prognosis; pT3 denotes extension through the muscularis into the subserosa or mesoappendix without involvement of the serosal surface; pT4a denotes mucin or epithelial cells reaching the visceral peritoneum, a finding that requires differentiation from intraoperative contamination or reactive tissue changes; and pT4b denotes direct invasion of or adhesion to adjacent organs. In contrast, M staging exerts greater prognostic influence: M0 indicates absence of peritoneal dissemination, M1a denotes acellular mucin only, and M1b denotes mucin deposits containing epithelial cells—M1b is strongly associated with disease progression and poorer outcomes (27–29).The PSOGI consensus, intended to complement AJCC staging, places greater emphasis on the histopathologic characteristics and grading of peritoneal disease. Low-grade mucinous peritoneal disease (formerly termed disseminated peritoneal adenomucinosis; DPAM or low-grade peritoneal carcinoma, G1) corresponds to peritoneal dissemination that may nonetheless contain low-grade epithelial cells (AJCC M1b). High-grade mucinous peritoneal carcinomatosis (PMCA; G2–G3), particularly when signet-ring cells are present (the most aggressive subtype), reflects worse biological behavior and has important implications for staging, prognosis, and postoperative management (30–32). For patients with LAMN confined to the appendix (pTis, M0), simple appendectomy followed by routine surveillance is generally sufficient. Recommended follow-up typically includes annual imaging and tumor marker assessment, with no further immediate treatment indicated (33, 34). Management of pT4a, M1a lesions—characterized by serosal breach with acellular mucin—is controversial: most centers favor close observation after appendectomy, whereas some centers may elect selective prophylactic cytoreductive surgery (CRS) with or without hyperthermic intraperitoneal chemotherapy (HIPEC) when additional high-risk features are present (23, 26). Reported overall survival with this aggressive approach has been approximately 75% at 5 years and 63% at 10 years (35). When peritoneal deposits containing epithelial cells are identified (any T, M1b; low-grade/DPAM), CRS plus HIPEC is recommended following complete macroscopic cytoreduction. In cases of peritoneal dissemination by high-grade adenocarcinoma or with signet-ring cell features (any T, M1b; PMCA/PMCA-SRC), CRS plus HIPEC remains the cornerstone of locoregional therapy but is commonly combined with systemic chemotherapy because of the more aggressive biology and poorer prognosis (36, 37). Postoperative surveillance for LAMN should be individualized according to pathological stage and risk stratification. For low-risk LAMN (pTis/pT3) with a very low probability of progression to PMP, a low-intensity follow-up strategy is reasonable: obtain a baseline contrast-enhanced CT at approximately 6 weeks postoperatively, with repeat CT at 18 months and 48 months, concurrently monitoring tumor markers (CEA, CA19-9, CA-125). Clinical follow-up may be performed annually, and in the absence of abnormalities imaging surveillance may be discontinued after 5 years (38). For high-risk LAMN (e.g., perforation, mucin dissemination, M1b disease, or mucin extending beyond the right iliac fossa), the risk of progression is appreciably higher (approximately 6%), and a structured surveillance program is recommended: annual contrast-enhanced CT scans with tumor marker assessments for the first 5 years, followed by CT every 2 years thereafter, extending overall surveillance to 10 years (23, 39).

In this case, laparoscopic exploration revealed significant inflammation, with the appendix indistinct from edematous cecum. Simple appendectomy risked anastomotic leak and recurrent abscess given compromised tissue integrity (40, 41). Additionally, a short or mobile cecum lacks sufficient appendiceal base support, increasing postoperative fistula risk if not completely resected (42). Extended ileocecal resection not only ensured removal of the acutely inflamed appendix but also secured clear margins around the neoplasm.

This case emphasizes the diagnostic and therapeutic value of laparoscopic exploration in patients with atypical, recurrent lumbar abscesses. In particular, when standard management fails to prevent recurrence and preoperative imaging suggests intra-abdominal communication, the possibility of rare hernias—such as lumbar herniation containing inflamed intra-abdominal organs—should be considered. Early surgical intervention not only facilitates definitive diagnosis but also enables timely resection of diseased tissues, abscess drainage, and prevention of further complications. Awareness of such uncommon presentations is crucial for surgeons and radiologists alike to avoid delayed diagnosis and ensure optimal outcomes.

## Data Availability

The original contributions presented in the study are included in the article/supplementary material. Further inquiries can be directed to the corresponding authors.
